# Opportunities and challenges of nanotechnology in the green economy

**DOI:** 10.1186/1476-069X-13-78

**Published:** 2014-10-07

**Authors:** Ivo Iavicoli, Veruscka Leso, Walter Ricciardi, Laura L Hodson, Mark D Hoover

**Affiliations:** Institute of Public Health, Catholic University of the Sacred Heart, Largo Francesco, Vito 1, 00168 Rome, Italy; National Institute for Occupational Safety and Health, Centers for Disease Control and Prevention, 4676 Columbia Parkway, MS C-14, Cincinnati, OH 45226 USA; National Institute for Occupational Safety and Health, Centers for Disease Control and Prevention, 1095 Willowdale Road, MS H2800, Morgantown, WV 26505 USA

**Keywords:** Green economy, Nanotechnology, Sustainable development, Occupational health, Safety, Environmental risk, Ecology, Engineered nanomaterials, Material science

## Abstract

In a world of finite resources and ecosystem capacity, the prevailing model of economic growth, founded on ever-increasing consumption of resources and emission pollutants, cannot be sustained any longer. In this context, the “green economy” concept has offered the opportunity to change the way that society manages the interaction of the environmental and economic domains. To enable society to build and sustain a green economy, the associated concept of “green nanotechnology” aims to exploit nano-innovations in materials science and engineering to generate products and processes that are energy efficient as well as economically and environmentally sustainable. These applications are expected to impact a large range of economic sectors, such as energy production and storage, clean up-technologies, as well as construction and related infrastructure industries. These solutions may offer the opportunities to reduce pressure on raw materials trading on renewable energy, to improve power delivery systems to be more reliable, efficient and safe as well as to use unconventional water sources or nano-enabled construction products therefore providing better ecosystem and livelihood conditions.

However, the benefits of incorporating nanomaterials in green products and processes may bring challenges with them for environmental, health and safety risks, ethical and social issues, as well as uncertainty concerning market and consumer acceptance. Therefore, our aim is to examine the relationships among guiding principles for a green economy and opportunities for introducing nano-applications in this field as well as to critically analyze their practical challenges, especially related to the impact that they may have on the health and safety of workers involved in this innovative sector. These are principally due to the not fully known nanomaterial hazardous properties, as well as to the difficulties in characterizing exposure and defining emerging risks for the workforce. Interestingly, this review proposes action strategies for the assessment, management and communication of risks aimed to precautionary adopt preventive measures including formation and training of employees, collective and personal protective equipment, health surveillance programs to protect the health and safety of nano-workers. It finally underlines the importance that occupational health considerations will have on achieving an effectively sustainable development of nanotechnology.

**Electronic supplementary material:**

The online version of this article (doi:10.1186/1476-069X-13-78) contains supplementary material, which is available to authorized users.

## Background

The “green economy” concept has been driven into the mainstream of policy debate by global economic crisis, expected increase in global demand for energy by more than one third between 2010 to 2035, rising commodity prices as well as the urgent need for addressing global challenges in domains such as energy, environment and health [1–3].

The term “green economy”, chiefly relating to the principles of sustainable development, was first coined in a pioneering 1989 report for the Government of the United Kingdom by a group of leading environmental economists [1]. The most widely used and reliable definition of “green economy” comes from the United Nations Environment Programme which states that “a green economy is one that results in improved human well-being and social equity, while significantly reducing environmental risks and ecological scarcities. It is low carbon, resource efficient, and socially inclusive” [4].

The green economy can also be viewed as a set of principles, aims and actions, which generally include: *(i)* equity and fairness, both within and between generations, *(ii)* consistency with the principles of sustainable development, *(iii)* a precautionary approach to social and environmental impact, *(iv)* an appreciation of natural and social capital, through, for example, the internalisation of external costs, green accounting, whole-life costing and improved governance, *(v)* sustainable and efficient resource use, consumption and production, and *(vi)* a need to fit with existing macroeconomic goals, through the creation of green jobs, poverty eradication, increased competitiveness and growth in key sectors [3–7].

The green economy concept can indeed play a very useful role in changing the way that society manages the interaction of the environmental and economic domains. In this context, nanotechnology, which is the manipulation of matter in the dimension of 1 to 100 nm, offers the opportunity to produce new structures, materials and devices with unique physico-chemical properties (i.e. small size, large surface area to mass ratio) to be employed in energy efficient as well as economically and environmentally sustainable green innovations [8–12].

Although expected to exert a great impact on a large range of industrial and economic sectors, the sustainability of green nano-solutions is currently not completely clear, and it should be carefully faced. In fact, the benefits of incorporating nanomaterials (NMs) in processes and products that contribute to outcomes of sustainability, might bring with them environmental, health and safety risks, ethical and social issues, market and consumer acceptance uncertainty as well as a strong competition with traditional technologies [13].

The present review examines opportunities and practical challenges that nano-applications pose in addressing the guiding principles for a green economy. Examples are provided of the potential for nano-applications to address social and environmental challenges, particularly in energy production and storage thus reducing pressure on raw materials, clean-up technologies as well as in fostering sustainable manufactured products. Moreover, the review aims to critically assess the impact that green nanotechnology may have on the health and safety of workers involved in this innovative sector and proposes action strategies for the management of emerging occupational risks.

### The potential nanotechnology impact on green innovations

Green nanotechnology is expected to play a fundamental role in bringing a key functionality across the whole value chain of a product, both through the beneficial properties of NMs included as a small percentage in a final device, as well as through nano-enabled processes and applications without final products containing any NMs [13, 14]. However, most of the potential green nano-solutions are still in the lab/start-up phase and very few products have reached the market to date. Further studies are necessary to assess the applicability, efficiency and sustainability of nanotechnologies under more realistic conditions, as well as to validate NM enabled systems in comparison to existing technologies. The following paragraphs will describe the potential fields of application for green nanotechnology innovations.

#### Nanomaterials for energy conversion

One of the most interesting and most flexible renewable energy technologies is the direct conversion of sunlight into electric power: the photovoltaic effect [8, 15]. Carbon NMs, including C_60_ fullerenes [16, 17], carbon nanotubes (CNTs) [18, 19] and graphene [20, 21] have been studied as extremely efficient electron acceptors in polymer and quantum dot solar cells [8]. Relatively new, dye sensitized solar cells are of great promise. In these devices, a nanocrystalline mesoporous titanium dioxide (TiO_2_) film, with a monolayer of the charge transfer dye attached to its surface, is pasted on a transparent conductive substrate [22, 23]. The large NM surface area for dye chemisorption and the short charge migration length underlie their power conversion efficiency [24, 25].

In addition to solar cells, nanotechnology has made big impact on fuel cells, devices able to convert chemical energy directly into electricity [24]. Nano-porous metals with high surface area, low specific densities and rich surface chemistry, can be highly efficient electro-catalysts for the critical electrode oxidation/reduction reactions in fuel cells [26, 27]. Platinum nanoparticles (Pt-NPs) have been regarded as the best cell catalyst, although the Pt-based electrode suffers from time-dependent drift and carbon monoxide deactivation [28]. In this regard, nano-sized multi (bi-tri)-metallic Pt alloys have been the object of further exploration because of their higher electro-catalytic activities and greater resistance [29, 30]. Interestingly, CNTs and graphene, initially used in fuel cells as attractive materials for catalyst supports with the aim to lower precious-metal loading, enhance catalyst activity and durability, are currently studied also as metal-free catalysts in fuel cells [31–33]. Their advantages rely on high surface area, mesoporosity, good electrical conductivity, stronger mechanical strength, light weight and superb corrosion-resistance [27].

Another important future energy option is the hydrogen gas as an endless source of clean fuel for many applications [34]. Semiconductor NMs, e.g. TiO_2_ and cadmium sulfide nanostructures, have been studied as efficient catalysts for water conversion into oxygen and hydrogen [35–37]. Moreover, nano-structured carbons, metal-organic frameworks and polymers [38–41] as well as metal hydrides and related complex hydrides [42, 43] are examples of investigated NMs for hydrogen storage and transportation for high hydrogen capacity and minimal deterioration during hydrogenation.

#### Nanomaterials for energy storage

Nanotechnology may have a profound influence on electrical storage technologies, i.e. batteries and electrochemical supercapacitors [44]. Redox-based supercapacitors with nano-structured electrode materials have shown the potential to combine the high energy density of conventional batteries with the high power capabilities of electrostatic capacitors at the lab scale. Mixed metal oxides, e.g. ruthenium oxide (RuO_2_), manganese oxide (MnO_2_), magnetite (Fe_3_O_4_) [45–47], CNTs [8, 48], graphene [49, 50] and carbon-metal oxide composites [51] have been investigated as electrode NMs aimed at a high specific capacity and rate capability [52, 53].

Concerning rechargeable lithium batteries, the energy densities and the performances of these devices largely depend on the physical and chemical properties of the electrode material [54]. In this regard, the reduced dimensions and high surface area of NMs increase the rate of electron transport and the electrode-electrolyte contact, respectively, while the nano-structure itself provides facile strain relaxation and resistance to fracture [24]. For anode applications, CNTs [8, 55, 56], a series of graphene-based nanostructures [21, 57, 58] and silicon nanowires [59] have been studied as promising host-high capacity materials and conductive additives. While emerging interests has been focused on metal oxide NMs, e.g. SnO_2_; TiO_2_ or LiFePO_4_-NMs, for anode or cathode applications [60–63].

#### Nanomaterials for water clean-up technologies

Nanotechnology-enabled water and wastewater treatment promises not only to overcome major challenges faced by existing treatment technologies, but also to provide new treatment capabilities that could allow economic utilization of unconventional water sources to expand the water supply [64]. Interesting applications may include the incorporation of functional NMs, such as metal-oxide NPs (aluminium oxide, TiO_2_ and zeolite) [65–67], anti-microbial NMs (silver-NPs (Ag-NPs) and CNTs) [68] and photocatalytic NMs (bimetallic-NPs, TiO_2_) [69, 70] into membranes in order to improve their permeability, fouling resistance, biofilm control, mechanical and thermal stability, as well as to provide pollutant degradation and self-cleaning ability [71]. Moreover, CNTs, fullerene and metal-based nano-adsorbents may offer significant improvement in the adsorption capacity of organic molecules, metal ions and heavy metals [72–75]. Interestingly, due to the NM-unique electrochemical, optical, and magnetic properties, active research is going on developing nano-enabled pathogen sensors, both cells or biomolecules [76, 77].

#### Nanomaterials for construction industry

Manufactured NMs and nanocomposites offer great opportunities in the construction and related infrastructure industries [78]. Strength, durability, and lightness of various materials [79, 80], as well as heat-insulating, self-cleaning, fire-retardant, anti-fogging and sensing structural health properties may be improved or provided *de novo* by NMs [81, 82]. Thus, CNTs, SiO_2_, TiO_2_, Fe_2_O_3_, and magnetic nickel-NPs can remarkably improve mechanical durability, compressive and flexural strength of cement products [83–86]. Highly water repellent coatings incorporating silica, alumina-NPs and hydrophobic polymers are proper to be used for wood [87]. The use of TiO_2_-NPs in glasses leads to the so-called self-cleaning technology due to their photo-catalytic and anti-fouling properties [81, 88]. Fire protective glass is obtained using silica (nano)layers, which may also function as antireflection coatings for exterior light in order to contribute to energy and air conditioning conservation [78, 89]. Ag-NPs can be embedded in paint to inactivate pathogenic microbes and provide antimicrobial properties to surfaces (e.g. hospital walls) [90].

#### Other nano-enhanced green applications

Several other sustainable nanotechnology applications have been investigated [91]. Nanoporous zeolites may be used as a slowly releasing carrier of fertilizers or as a permanent water reservoir due to their property to hold water molecules that may help plants to withstand dry spell [92]. Innovative NM properties may be useful in developing new-packaging to obtain films with good exfoliation, barrier, fireproofing and mechanical properties [93]. This application may permit to increase the shelf life of the food and its safety for consumers, especially in regions where cooling is not easily available. Nano-sensors can improve the quality and reduce the cost of continuous environmental monitoring [91, 94].

### Occupational health and safety considerations

The unique properties of nano-scale materials have made them attractive for a number of innovative, sustainable, green applications. A summary of the example relationships among the guiding principles for a green economy, opportunities and practical challenges for nano-applications in a green economy is presented in Table 1.

**Table 1:** **Example relationships among guiding principles for a green economy and the opportunities and challenges for nano-applications**

Guiding principles for a green economy (based on the proposals of ref.[127])	
(P1)	Is a means for achieving sustainable development;
(P2)	Creates decent work and green jobs;
(P3)	Improves governance and the rule of law – by being inclusive; democratic; participatory; accountable; transparent and stable;
(P4)	Is equitable, fair and just – between and within countries and between generations;
(P5)	Reduces poverty, and increases well-being, livelihoods, social protection, and access to essential services;
(P6)	Protects biodiversity and ecosystems;
(P7)	Is resource and energy efficient;
(P8)	Respects planetary boundaries or ecological limits or scarcity;
(P9)	Uses integrated decision making;
(P10)	Internalizes externalities;
(P11)	Measures beyond gross domestic product indicators and metrics
**Example opportunities for nano-applications in a green economy (and the related principles)**	
**Energy conversion and storage**	-Smart energy nanotechnology can improve power delivery systems to be more efficient, reliable and safe (P1, P2, P5).
	-Nano-devices may trade on renewable energy produced through naturally replenished resources, i.e. sunlight and wind. This may reduce fossils as energy resources and the impact for the greenhouse gas emissions balance (P3, P4, P5, P6, P7, P9).
	-Energy efficient nanotechnology requires less energy to perform the same function - getting more use out of the already created energy (P7, P8, P10).
**Water clean-up technologies**	-Design nano-enabled infrastructure necessary to manage water and keep it clean is inextricably linked to prospects for economic development and better livelihood conditions (P1, P2).
	-Access to clean water and adequate sanitation is a basic human right and is critical to the alleviation of poverty (P3, P4, P5).
	-Investment in infrastructures and considerable greening of water policies are necessary to reduce the cost to face water shortages (P8, P9, P10, P11).
**Construction industry**	-Nanotechnology aims to increase the efficiency buildings use resources - energy, water, and materials - while reducing building impacts on environment and human health through better siting, design, construction, and removal (P1, P2, P6, P7, P8, P10, P11).
	-NMs applied to the surfaces of structural elements of the buildings can contribute to environmental cleaning by photo-catalytic reactions (P1, P2, P6, P7, P8).
**Other applications**	-Nano-enabled applications may provide a slow release and dosage of fertilizers and an efficient water reservoir for plants. This may contribute to a greater agricultural productivity, especially in countries with prolonged dry spells (P1, P2, P4, P5).
	-Nano-packaging - with improved barrier and mechanical properties - may allow a longer safe storage of food, especially in regions where cooling is not easily available (P2, P4, P5, P8).
	-Nano-sensors may improve the quality and reduce the cost of continuous environmental monitoring. Nano-remediation of environmental pollution may exceed conventional methods in efficiency and speed (P1, P2, P6, P7).
**Practical challenges for nano-applications in a green economy**	
**Technical**	-Efficient synthetic pathways must be developed to obtain NMs “safe by design” (e.g. through green chemistry; optimized reaction chemistry; minimized energy consumption and costs; employment of benign feed stocks and reagents; avoidance of hazardous substances and pollutants);
	-Analytical methods must be developed to obtain a reliable nanomaterial characterization and tools to detect, monitor and track NMs in the environment and biological media.
**Biological**	-Biological impact must be determined for NM primary and acquired physico-chemical properties (size, surface area, chemical composition, protein corona as a nano-bio interaction) on ecosystems, as well as in *in vitro* and *in vivo* models;
	-The “life-cycle” impact must be assessed for NMs on the environment and biological systems: NMs emitted from production processes, or released from nano-enabled devices during their assembly, use, recycling or disposal.
**Health and safety**	-NM key health effects must be defined: e.g. pulmonary toxicity, genotoxicity and carcinogenicity.
	-Information must be developed on the potential toxicity of NMs available for employers and workers involved in NM research and developmental areas, as well as in nano-enabled device manufacture, assembly, application and disposal, avoiding dispersion of essential information.
	-A highly skilled workforce must be built and sustained, that is well trained to face emerging risks as well as known physico-chemical risks in new situations and also trained to avoid accidents.
**Public and occupational policies**	-Participation of scientific, governmental, industry and workforce representatives must be pursued for the processes of opinion forming, education and decision making in shaping green nanotechnology.
	-Nano-green jobs must redirect current path of environmental decline and create economic opportunity, strengthening local urban and rural communities.
	-The green economy policies must balance nanotechnology environmental, societal, occupational and health promotion benefits, with commercialization costs and risks.
	-Companies involved in green-nanotechnology innovations must plan a precautionary risk management approach by identifying actual risks, planning/implementing control measures and risk communication.

**Figure 1:**
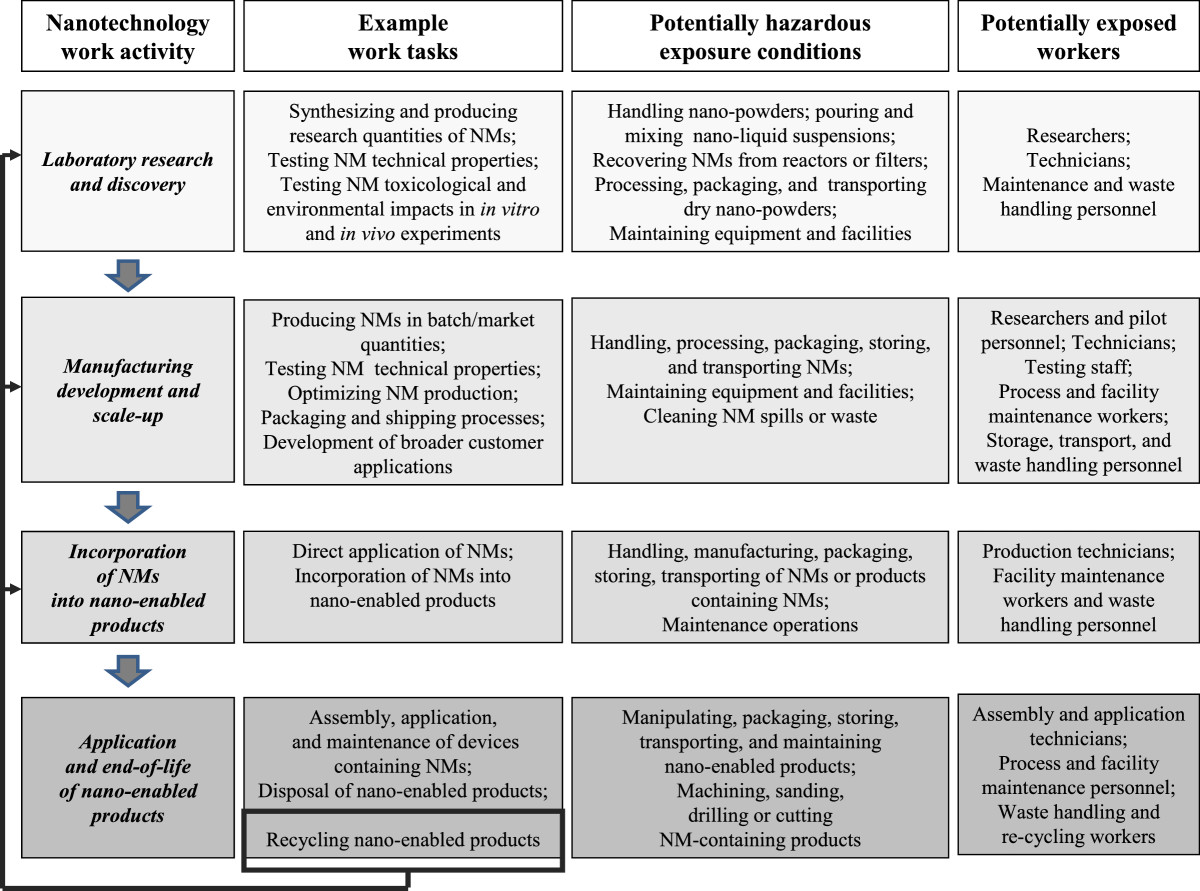
**An analysis of potentially hazardous exposure conditions for workers involved in nanotechnology activities.** Legend: Note how the recycling of nano-enabled products over time may result in changes in the composition of workplace exposures.

In this context according to the proposed principles for green economy, it is important that society, scientific community and industry take advantage of opportunities of nanotechnology while overcoming its practical challenges. However, not all revolutionary changes are sustainable per se and a cautious assessment of the benefits addressing economic, social and environmental implications, as well as the occupational health and safety impact is essential [95, 96]. This latter aspect, in particular, should be carefully addressed, in consideration of the expected widespread use of nanotechnology and the consequent increasing likelihood of NM exposure in both living and occupational environments. Moreover, difficulties in nano-manufacturing and handling; uncertainty concerning stability of nano-innovations under aggressive or long-term operation (i.e. in the case of supercapacitors with nano-structured electrode materials or nano-enabled construction products); the lack of information regarding the release and fate of NMs in the environment (i.e. NMs released from water and wastewater treatment devices) as well as the limited knowledge concerning the NM toxicological profile, even further support the need for a careful consideration of the health and safety risks derived from NM exposure.Importantly, as shown in Figure 1, a number of potentially hazardous exposure conditions can be expected for workers involved in nanotechnology activities. In fact, NMs may have significant, still unknown, hazards that can pose risks for a wide range of workers: researchers, laboratory technicians, cleaners, production workers, transportation, storage and retail workers, employees in disposal and waste facilities and potentially, emergency responders who deal with spills and disasters of NMs who may be differently exposed to these potential, innovative xenobiotics.

In this scenario, responsible green-nanotechnology development requires careful consideration of the possible lifecycle impact of NMs on the health of workers [97]. The earliest exposures to NMs may occur for those workers conducting discovery research in laboratories involved in designing, synthesizing and testing the usefulness of NMs in a variety of applications as well as in determining their toxicological and environmental impacts [98]. Workers in start-up and manufacturing companies involved in pilot processes can be exposed during several phases such as handling nano-powders, pouring or mixing nano-liquid solutions, recovering products from reactors or filters, conducting maintenance of equipment or rooms, cleaning spills or waste NMs or during processing, packaging and transporting of dry powder [99, 100]. The variability in composition, morphology and purity of NMs due to the developing methods of production, their dynamic behavior once dispersed in the workplace (e.g. agglomeration, aggregation, interaction with other airborne particles) and the large number of parameters required for their complete characterization, make NM exposure assessment a challenging issue [78, 91]. Furthermore, it may be extremely important to assess the exposure to “fresh” NMs, directly emitted from production processes, or “aged” NMs released during handling of packaged NMs, when assembling or maintaining nano-enhabled devices or released through wear and tear of nano-products [101–104]. Ambient aging conditions, may cause NMs to undergo physical, chemical, and/or biological transformations that may change their properties and consequently their biological reactivity [78]. All these aspects impact the technical difficulties to routinely monitor, measure and characterize NMs in workplace settings principally related to the lack of easy to use instruments as well as to the difficulty in defining dose metric parameters, other than traditional mass, which may better reflect the NM biologically effective dose in the nano-toxicological field [102, 105, 106].

Workers should also be aware of their potential exposure to NMs or products containing them during the disposal stage [100]. Laboratories, industries, and disposal of products containing NMs may produce new forms of waste that could challenge current waste management, product re-use and recycling efforts. When addressing the issue of occupational NM exposure in these contexts, a variety of factors should be carefully considered. The intrinsic potential of different devices to release NMs, the possible disposal pathways for specific nano-waste, (e.g. waste-water, landfill, incineration or recycling), the bioavailability and persistence of NMs, and subsequent effect in and across the disposal media such as air, soil and water all need specific attention [100].

Nano-enhanced technologies may lead to “emerging occupational and safety risks” principally related to the exposure to candidate NMs whose toxicological behaviors and mechanisms of biological reactivity are still under research. In this regard, scientific concerns raised in consideration of the preliminary results demonstrating the pro-oxidant and pro-inflammatory action of several types of engineered NMs *in vitro*[107–109] and also their ability to induce alterations in organs such as lung, cardiovascular and central nervous system following acute to chronic periods of treatment *in vivo*[110–112]. Recent evidence of the occupational carcinogenic potential of inhaled TiO_2_-NPs [113], we previously mentioned as components of innovative solar cells or of self cleaning construction products, the uncertainty concerning whether some types of CNTs, which may find widespread employment in a series of energy conversion and storage devices, may be carcinogenic [114], as well as the possibility of not recognizing long latency hazards of innovative materials heighten these concerns. Therefore, new demanding green nanotechnologies will need a highly skilled workforce, well trained in specific skills necessary to perform these jobs, as well as in recognizing risks and taking occupational safety and health measures to reduce risks induced by new processes and products. This ambitious aim will require large scientific efforts to overcome the current lack of knowledge concerning NM hazardous properties as well as governmental engagement and empowerment of workers aimed to “assure/make sure” workforce education and regulation in order to reach suitable employee expertise, good workplace practices and adequate health protection. Importantly, a comprehensive approach to prevention and protection strategies in green nanotechnologies should take into account also the relevance that a careful assessment and management of “known” physico-chemical risks, intrinsically related to the manufacture, installation, maintenance and disposal of nano-enabled products, may have for the health and safety of workers.

### Green nanotechnology: risk assessment, management and communication

The newness of nano-applications in green fields, together with concerns regarding the potential impact of NMs on the health and safety of workers, urgently require scientific, technological and governmental efforts to actively manage risks for the workforce. This means to identify actual risks derived from NM exposure in workplace (risk assessment), to plan/implement control measures (risk management) and to communicate the plan. Overall, these steps, whose critical aspects will be discussed in the following sections, aim to prevent workers to be harmed and society deprived of the timely realization of all the benefits of the nanotechnology.

#### Risk assessment

Risk assessment of NMs notably includes the same steps used for the risk assessment of other types of chemicals [102]. These are: hazard identification; hazard characterization with an emphasis on defining critical target organs and dose-response relationships; assessment of exposure for different scenarios and a synthesizing step risk characterization [115].

Unfortunately, the NM risk assessment process still suffers from a lack of toxicological data on a wide variety of NMs. Therefore, future research should be aimed to systematically improve the understanding of metrics, such as size, surface area, functionalization or particle number concentration, that may be responsible for NM toxicity. The toxico-kinetic and toxico-dynamic behaviour of NMs in biological systems, including the influences exerted by the protein corona formation, as the result of a dynamic nano-bio interaction, should be deeply investigated. Moreover, the definition of key health effects such as pulmonary toxicity, genotoxicity or carcinogenicity in conditions of long-term, low-dose exposure resembling realistic scenarios, require attention [116]. Exposure assessment remains a fundamental condition for the characterization of occupational NM risks [106, 114, 115]. Thus, efforts should be made to overcome practical barriers related to the newness of green-NM exposure scenarios, the inconsistencies over how to identify and classify NMs, and the questions about metrics for health related-sampling and practical instrumentation [117]. Moreover, biological monitoring studies, are also important to define possible biomarkers of NM exposure and effect to be prospectively tested and validated in workplace settings and used for an adequate evaluation of occupational risks [118].

#### Risk management

The ultimate goal of risk assessment is to provide quantitative predictions of given risks enabling their evidence-based management [102]. However, vast uncertainty about hazards, exposures, and risks in the emerging green nanotechnology field, make it imperative to adopt a dynamic-precautionary management approach before all of the evidence is completed. This means that risk management strategies and guidance will be changing and continuously evaluated, improved, and verified as risk information becomes more substantial [97].

To effectively manage potential green nanotechnology related risks, a risk management plan including a hierarchy of controls should be emphasized [119]. The first step in developing such a plan is to determine which workers may have potential exposures, measuring these exposures and identifying how the exposure may vary depending on the job task (as illustrated in Figure 1). Potential worker exposures should be managed using the hierarchy of controls starting with elimination of the hazard, adopting a green chemistry through the substitution with a non-hazardous or less hazardous alternative (such as modifying the molecule if possible), and introduction of engineering controls such as enclosed systems, local exhaust ventilation, engineering hood or pressure differentials [120]. These steps should be followed by administrative controls, including training programs through which companies communicate to workers information sufficient to understand the nature and routes of potential NM workplace exposure, possible risks, adequate job procedures, preventive and protective measures and policies adopted. In this context, it may be important to overcome the frequently insufficient or inadequate information present on safety data sheets [121]. The use of personal protective equipment (PPE), such as respiratory and eye protection, lab coats and gloves, should be addressed as the final step for exposure control because the use of PPE puts the responsibility on the employee instead of the employer [119]. Among these primary preventive measures, greater efforts should also be targeted to pursue the ambitious attempt to adopt sustainable practices, throughout the lifecycle and value chain of NMs, which may allow to design production processes able to reduce exposure and to obtain less toxic NMs, ”safe by design” [120]. Good business practice includes planning for controlling possible exposure scenarios during the design process as this is frequently less expensive than retrofitting existing process equipment. Aligning safety goals with business goals can improve the profitability of the business by protecting the employee skill, experience and knowledge; reducing production delays; and reducing any costs associated with employee injuries.

Occupational health surveillance can be a useful component of a NM risk management plan, which includes elements of hazard and medical surveillance [114, 117]. Monitoring of health outcomes or biological changes including medical surveillance of the effects at group and individual levels is part of an occupational health surveillance program [122, 123]. Medical surveillance may include: *(i)* an initial medical examination and collection of medical and occupational histories; *(ii)* periodic medical examinations at regularly scheduled intervals, including specific medical screening tests when warranted; *(iii)* more frequent and detailed medical examinations as indicated on the basis of findings from these examinations; *(iv)* post-incident examinations and medical screening following uncontrolled or non-routine increases in exposures such as spills; *(v)* worker training to recognize and report symptoms of exposure to a given hazard, *(vi)* a written report of medical findings; and *(vii)* employer actions in response to identification of potential hazards and risks to health [124].

Epidemiological research may be useful to enhance the impact of occupational health surveillance through the periodic analysis of aggregated data in order to identify patterns of worker health that may be linked to work activities and practices [114, 121, 125]. Exposure registries may be useful in setting the stage for this kind of research. Registries are important to enumerate and identify exposed individuals, to provide them with adequate information and guidance as well as with primary or secondary prevention measures concerning potential NM exposure risks [117].

#### Risk communication

Risk communication, is essential for the healthy innovation and sustainable development of green nanotechnology in view of a general public transparency [126]. In this context, risk communication should become effective in terms of making available complex technical and health information in language accessible and understandable to the occupational and general population. Importantly, researchers, regulatory scientists, representatives of the workforce, industry and governmental authorities should be actively engaged in facing a dialogical pro-active communication of the potential nanotechnology risks with the aim to form adequate perceptions and attitudes. This appears extremely important to assure the spread, also promoted by *mass media,* of appropriate information regarding benefits and challenges of nanotechnology, protecting public and personnel opinion from both unrealistic hopes and excessive awareness in this regard.

## Conclusions

Green nanotechnology aims to exploit the attractive physico-chemical properties of NMs in a number of green-innovative applications that are energy efficient as well as economically and environmentally sustainable, expected to exert an exciting impact on a large range of economic sectors. These solutions may offer the opportunities to reduce pressure on raw materials trading on renewable energy, to improve power delivery systems to be more reliable, efficient and safe as well as to use unconventional water sources or nano-enabled construction products therefore providing better ecosystem and livelihood conditions [127]. However the opportunities offered by NMs in green economy solutions, should be balanced with a number of practical challenges, critical environmental and social issues, as well as with human health and safety concerns. In particular, NMs may have significant, still unknown, hazardous properties related to their unique physico-chemical properties, that can pose risks for a wide range of employees potentially exposed through the overall lifecycle of NMs. Therefore, scientific research, technological, governmental and workforce efforts should be focused to deeply define the hazardous impact of NMs with the aim to reach an adequate risk assessment. This would provide helpful information and guidance to adopt appropriate preventive and protective measures in a comprehensive risk management program both for the general and occupationally exposed populations.

Overall, green nanotechnology should not only provide green solutions, but should also “become green” in terms of the attention paid to occupational safety and health. In this context, a full democratic discussion between expertise should be pursued to carefully balance the benefits of green nanotechnology and the potential costs for the society, particularly in terms of environmental, public and occupational health. This careful consideration will maximize environmental and societal benefits, health gains and cost savings and will increase the likelihood of further investment and sustainable development of this promising technological field.

## Authors’ original submitted files for images

Below are the links to the authors’ original submitted files for images. Authors’ original file for figure 1

## Abbreviations

Ag-NPs: Silver nanoparticles
CNTs: Carbon nanotubes
NMs: Nanomaterials
NPs: Nanoparticles
PPE: Personal protective equipment
Pt-NPs: Platinum nanoparticles
TiO_2_: Titanium dioxide.
