# Supplementary figures and images for: Opportunities and challenges of nanotechnology in the green economy
Source: Environ Health. 2014 Oct 7;13:78. doi: 10.1186/1476-069X-13-78 (PMC4201727; doi:10.1186/1476-069X-13-78)

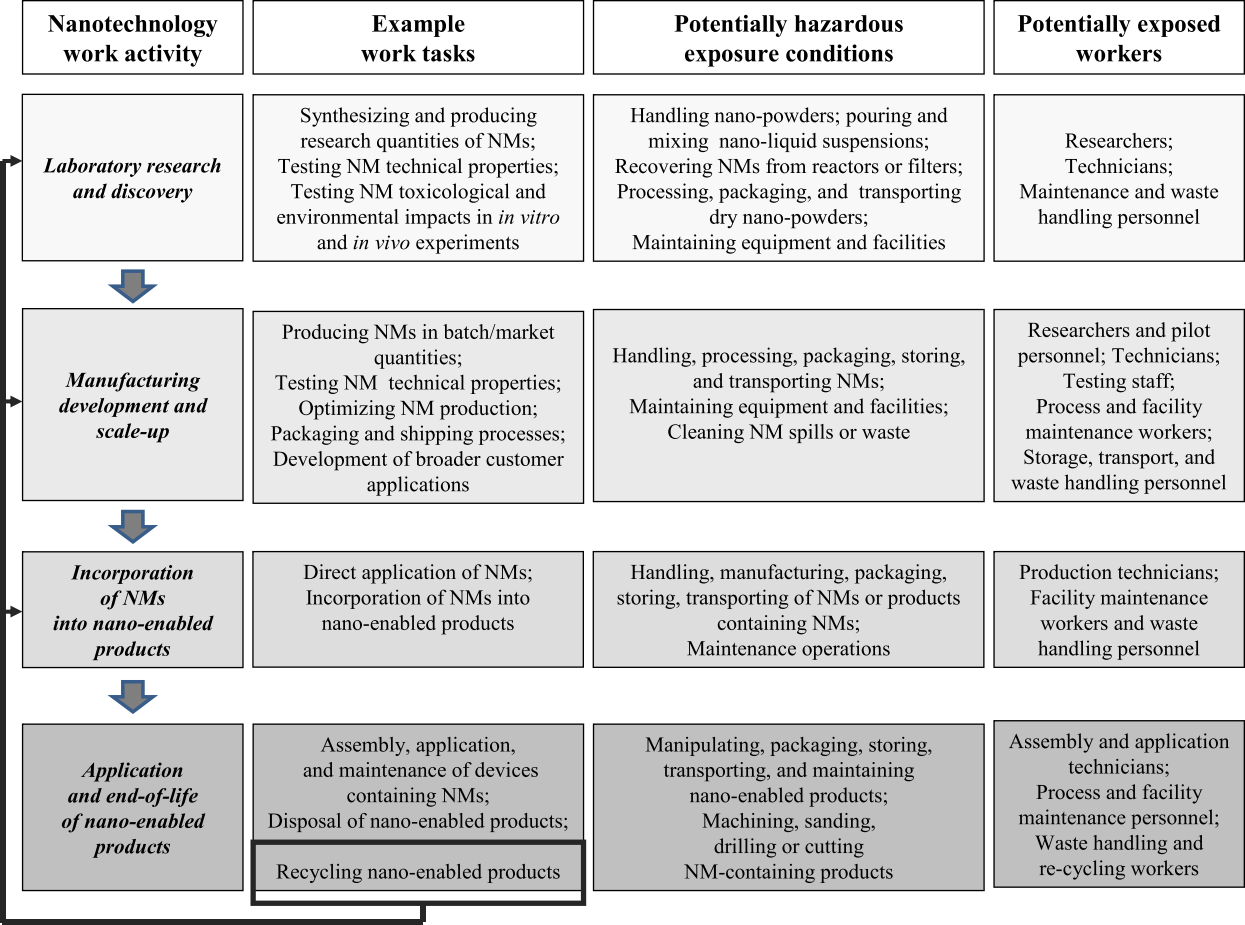

Supplement: Supplementary file 1 — Authors’ original file for figure 1 [file 12940_2014_786_MOESM1_ESM.pdf]
